# Advances in salt tolerance molecular mechanism in tobacco plants

**DOI:** 10.1186/s41065-020-00118-0

**Published:** 2020-02-24

**Authors:** Haiji Sun, Xiaowen Sun, Hui Wang, Xiaoli Ma

**Affiliations:** 1 School of Life Science, Shandong Normal University, Jinan, 250014 China; 2 Central laboratory, Jinan Central Hospital Affiliated to Shandong University, Jinan, 250013 China

**Keywords:** Salt tolerance, Transgenic technology, Gene, Tobacco

## Abstract

Tobacco, an economic crop and important model plant, has received more progress in salt tolerance with the aid of transgenic technique. Salt stress has become a key research field in abiotic stress. The study of tobacco promotes the understanding about the important adjustment for survival in high salinity environments, including cellular ion transport, osmotic regulation, antioxidation, signal transduction and expression regulation, and protection of cells from stress damage. Genes, which response to salt, have been studied using targeted transgenic technologies in tobacco plants to investigate the molecular mechanisms. The transgenic tobacco plants exhibited higher seed germination and survival rates, better root and shoot growth under salt stress treatments. Transgenic approach could be the promising option for enhancing tobacco production under saline condition. This review highlighted the salt tolerance molecular mechanisms of tobacco.

## Background

Abiotic stress is the most harmful factor concerning the growth and productivity of crops worldwide, leading to enhanced accumulation of osmolytes, reduced photosynthesis, closure of stomata, and induction of stress-responsive genes [1–5]. Salt stress is one of the major abiotic stresses that have been related to the significant economic impact caused by the loss of arable land and the decline of agricultural productivity [6–8]. Salt stress caused the crop damages via ion balance, osmotic regulation and removal of reactive oxygen species [9–12]. Inducing these pathways through short-term exposure to low-salt stress, a process known as salt adaptation, can improve plant resistance to salt [13–15]. However, tolerance to soil salinity levels varies between plant species.

Tobacco (*Nicotiana tabacum* L.) is one of the main industrial crops and is widely grown in many countries. Tobacco is forming complex defenses to resist salt stress that rely on a variety of mechanisms [16–19]. Generally, salt stress in tobacco can be divided into ion toxicity, such as destroying plasma membrane structure, hindering the absorption of mineral elements, etc. and the secondary stress effect, such as oxidative stress, drought stress, etc. [20, 21]. In this review, the recent advances on the mechanism of salt tolerance in tobacco were summarized in order to provide data for the study of salt tolerance and the adjustment of planting layout in tobacco.

### Ion transport genes related to tobacco salt tolerance

The activities of ion transporters or antiporters localized in the plasma membrane and vacuolar membrane are essential for tobacco growth and development [22–24]. Intracellular regionalization of toxic ions using specific transporter proteins is a key pattern used by tobacco to maintain a moderate cytosolic K^+^/Na^+^ ratio in the cytosol. The high-affinity potassium ion transporter protein selectively absorbs K^+^ from the environment to balance the ratio of Na^+^/K^+^ in cells and prevent the toxicity of excessive Na^+^ content to cells [25–28]. Constitutive expression of potassium transporter OsHAK5 in cultured-tobacco BY2 (*Nicotiana tabacum* cv. Bright Yellow 2) cells enhanced the accumulation of K^+^ but not Na^+^ in the cells during salt stress and conferred increased salt tolerance to the cells, suggesting that the plasma-membrane localized Na^+^ insensitive K^+^ transporters could be used as a tool to enhance salt tolerance in tobacco [29]. Na^+^ transporter protein (SKC) can transport Na^+^ exclusively, but does not participate in the transport of other cations such as K^+^, and plays an important role in resisting abiotic stress [30–32]. The survival rate and root length of SbSKC1 transgenic tobacco plants under NaCl stress were significantly higher than those of the control [33]. The activities of superoxide dismutases (SOD), catalase (CAT), and pero-xidase (POD) enzymes were increased, and the salt tolerance of transgenic tobacco plants was strengthened [34].

Na^+^/H+ reverse proteins are mainly located in the vacuole membrane and cytoplasmic membrane, which are called vacuolar Na+/H+ reverse transporter (V-type and P-type) [35]. Na^+^/H^+^ antiporters (NHXs) are integral membrane transporters that catalyze the electro-neutral exchange of K^+^/Na^+^ for H^+^ and are implicated in cell expansion, development, pH/ion homeostasis and salt tolerance [36, 37]. Different NHX isoforms have been overexpressed in variety of plant species showed substantial salt tolerance. NHX1 had functions in regulating the pH in the vacuole and cellular ROS level, which could prime the antioxidative system [38, 39]. *Arabidopsis* AtNHX1, the first tonoplast Na^+^/H^+^ exchanger identified in plants, mediates Na^+^/H^+^ exchange activity in plant vacuoles [40]. Overexpression of AtNHX confers salt tolerance in Arabidopsis plants and salt tolerance correlates with increased vacuolar Na^+^/H^+^ exchange activity and vacuolar sodium accumulation. LfNHX1 protein sequence showed high similarity with NHX1 homologs reported from other halophyte plants. The overexpression of LfNHX1 gene under CaMV35S promoter conferred salt and drought tolerance in tobacco plants [41, 42]. NbNHX1 silencing led to a lower pH in the vacuole and a lower cellular ROS level in N. benthamiana, which was coupled with a decreased NAD(P) (H) pool and decreased expression of ROS-responsive genes [43]. Overexpression of SeNHX1 intensified the compartmentation of Na + into vacuole under salt stress and improved the ability of eliminating ROS after pathogen attack, which then enhanced salt tolerance and disease resistance simultaneously in tobacco [44]. SeNHX1, AtNHX1, sbNHX1 and NbNHX1 transgenic tobaccos exhibited more biomass, longer root length, and higher Na+/H+ ratio under NaCl treatment, indicating enhanced salt tolerance [45].

### Osmotic regulation genes related to tobacco salt tolerance

Betaine is a water-soluble alkaloid in plants and has a strong affinity as an osmotic regulator [46]. The exogenous application of glycine betaine upregulates many proteins including PSII, Rubisco and superoxide dismutase when plants are subjected to NaCl stress [47, 48]. In general, the main synthetic pathway of betaine in plants is to produce betaine aldehyde catalyzed by choline monooxygenase (CMO) and then by betaine aldehyde dehydrogenase (BADH) [49]. The transgenic tobacco plants transferred by CMO gene of Salicornia salsa could grow normally under salt stress [50]. By introducing rice OsCMO gene into tobacco, it was found that the transgenic tobacco plants increased and the tolerance to salt stress increased [51]. Genetically engineered tobacco was established for the biosynthesis of glycine betaine in vivo and this tobacco showed increased tolerance of photosynthesis to salt stress.

Proline is small molecular organic compound that has been demonstrated to play a protective role in defending against high salinity stresses [52]. Under salt stress, plant tissues accumulate proline to alleviate the toxic effect of excessive ammonia on the organism, scavenge free radicals to protect the integrity of plasma membrane and regulate osmotic pressure to prevent the change of plasma membrane permeability [53]. Glutamic acid, the biosynthetic precursor of proline, mainly comes from glutamine synthase-glutamic acid synthase (GS-GOGAT). Plant glutamine synthase has many isoenzymes, which can be divided into cytosolic glutamine synthase (GS1) and plastid glutamine synthase (GS2) [54]. Over-expression of TaGS1/TaGS2 in tobacco could increase proline content, nitrogen use efficiency and salt tolerance under salt stress [55]. The transgenic tobacco plant over-expressing CsGSTs exhibited both drought and salinity stress tolerance [56].

### Reactive oxygen species (ROS) detoxification genes related to tobacco salt tolerance

Tobacco plants accumulate a large number of reactive oxygen species under stress [57]. ROS detoxification plays a protective role in response to salt stress by scavenging toxic radicals [58, 59]. Antioxidative defense systems include both non-enzymatic and enzymatic components, including superoxide dismutases (TaSOD) [60], monodehydroascorbate reductase (MDAR) [61], glutathione transferases (SbGST, SsGST) [62, 63], ascorbate peroxidases (SssAPX and PtcAPX) [64, 65]. They have been shown to play important roles in protecting against salt-induced oxidative stress. Gene engineering was used to express ROS scavenger factor to improve salt resistance in Tobacco plants [66].

The transcription of AhCuZnSOD gene in transgenic tobacco plants was up-regulated under abiotic stresses such as salt, drought, high salinity, cold and oxidative stress, which increased SOD activity and improved oxidative damage under abiotic stress [67]. When LetAPX gene was transferred into tobacco, the activity of APX in transgenic tobacco plants increased significantly, the germination rate of tobacco seeds increased, and the tolerance to salt stress increased [68]. 2-Cys peroxidoreductases has the ability to scavenge ROS in the chloroplast. Transforming the gene into tobacco can increase SOD activity, inhibit APX activity, enhance the stability of photosynthetic electron transport chain under high salt environment, and reduce the photoinhibition degree of PSII [69].

### Signal transduction genes related to tobacco salt tolerance

Studies have shown that transcription factors (TFs), such as AP2/ERF and WRKY, play an important role in abiotic stress and mediate the diversity of signal transduction processes induced by abiotic stresses such as salt, oxidation, cold and drought [70, 71]. AP2/ERF is a large group of plant-specific TFs and could be classified into four major subfamilies: the AP2, RAV, ERF, and Dehydration responsive element binding protein (DREB) subfamilies [72, 73]. Many ERF genes are reported to be involved in responses to salinity/drought. JcERF1 gene from *Jatropha curcas* was introduced into tobacco, which could enhance the salt tolerance of tobacco [74]. It was found that the salt tolerance of LchERF transgenic tobacco was improved. When ERF76 gene was introduced into tobacco, the germination rate, root length, fresh weight, SOD, POD activity and proline content of transgenic tobacco seeds increased under salt stress, and the salt tolerance of tobacco was improved [75]. DREB transcription factors involved in the progress of salt tolerance related genes in tobacco via plant stress response signaling [76].

WRKY transcription factors are new transcription regulators with highly conserved amino acid sequences at the N-terminal found in plants, which can specifically interact with TGAC sequences, regulate the expression of regulatory genes and functional genes with w-box elements in promoters, and play an important role in abiotic stress [77, 78]. In transgenic tobacco, overexpression of SpWRKY1, GhWRKY25, and TaWRKY44 promotes tolerance to salt and drought stress [79].

Zinc finger proteins comprise a family of transcription factors, mainly through the combination with Zn^2+^ to maintain a self forming “finger” structure domain, participating in the regulation of gene expression. The transcription factors of zinc finger proteins can be divided into C2H2, C2C2 and C2HC, among which C2H2 is the most abundant zinc finger protein in eukaryote genome [80, 81]. Citrus PtrZPT2–1 was introduced into tobacco, the osmotic adjustment solute increased and the hydrogen peroxide decreased, which enhanced the cold, drought and salt tolerance of tobacco plants [82].

In addition, calcium-dependent protein kinase synthesis pathways (CDPKs) play an important role in the downstream effect of calcium signaling. ZoCDPK1 gene can increase salt tolerance of tobacco, and the up regulation of ZoCDPK1 expression level in tobacco is related to RD21A and ERD1 genes related to stress [83, 84]. Mitogen-activated protein kinase (MAPK) cascades play key roles in the transduction of hormone signals, plant cytokinesis, pollen development, and biotic and abiotic stresses [85, 86]. The overexpression of the PtMAPKK4 enhanced the activity of antioxidant enzyme through an up-regulation of its expression, and the reduction of reactive oxygen species (ROS) could improve the plant tolerance of stress [87]. Furthermore, accumulating evidence suggests that miRNAs, key enzyme genes for sterol synthesis of Brassinol, hormonal regulation and resistance (R) genes play essential roles in both abiotic and biotic stress responses in tobacco plants [88–91].

## Conclusions

**Fig. 1:**
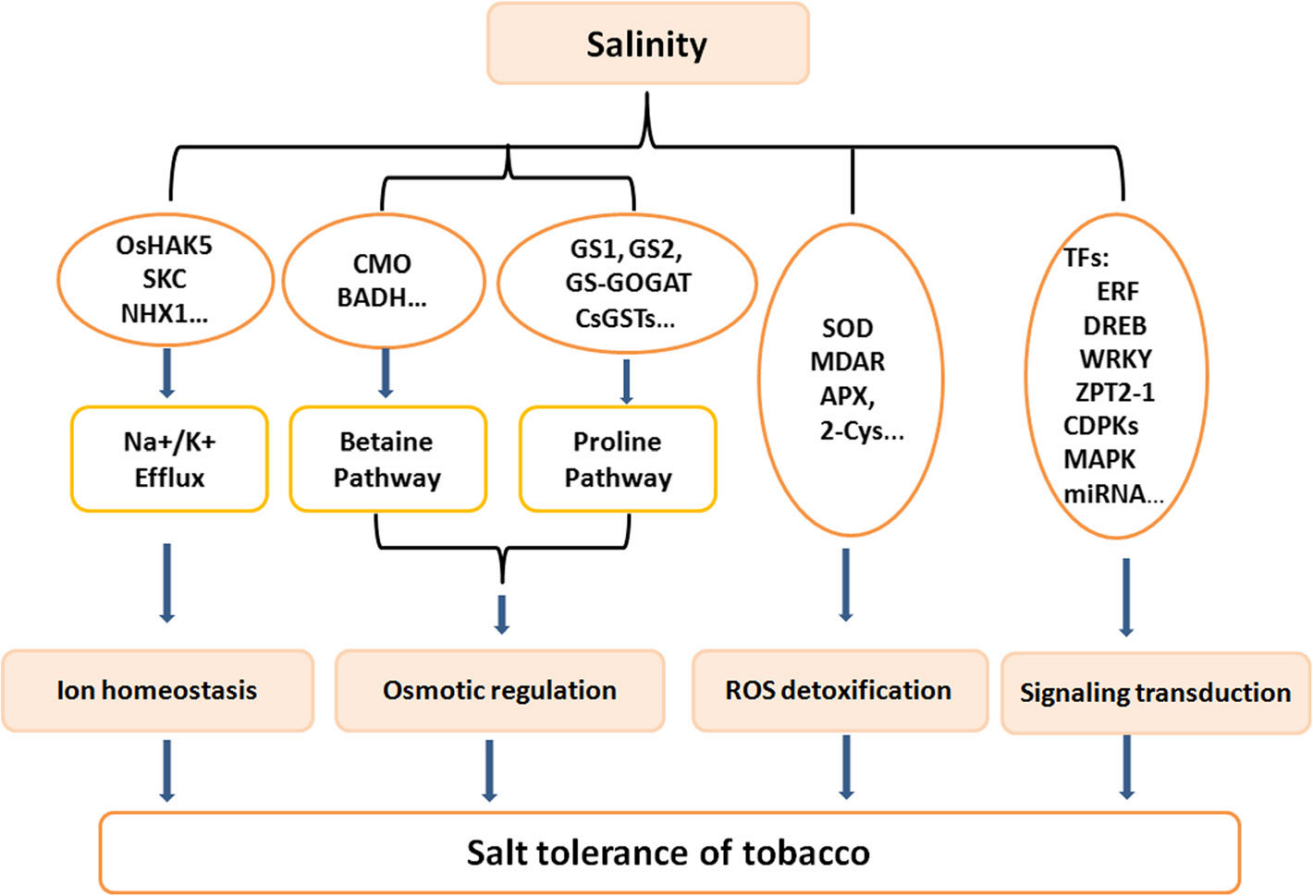
A proposed model of molecular mechanism of tobacco in response to salt stress

Salinity is the serious problem for agricultural productivity as many countries are facing this problem. Tobacco has recently been investigated as a potential model crop to adapt to salt stress via various strategies to cope with cellular ion homeostasis, osmotic pressure, antioxidation and signaling transduction regulation. Based on the studies focused on the molecular mechanisms associated with stress responses, a common set of proteins and regulatory pathways contribute to adaptation in tobacco plants (Fig. 1). A clearer idea of the mechanisms that contribute to salt tolerance in tobacco plants would facilitate their application in improving organismal tolerance to salinity stress. The combination of conventional and advance molecular technology is conducive to the study of salt-tolerant varieties.

## Data Availability

Not applicable.

## Abbreviations

APX: Ascorbate peroxidase
BADH: Betaine aldehyde dehydrogenase
CAT: Catalase
CDPKs: Calcium-dependent protein kinase
CMO: Choline monooxygenase
DREB: Dehydration responsive element binding protein
ERF: Ethylene response factor
GS1: Cytosolic glutamine synthase
GS2: Plastid glutamine synthase
GS-GOGAT: Glutamine synthase-glutamic acid synthase
GST: Glutathione transferase
MDAR: Monodehydroascorbate reductase
POD: Pero-xidase
ROS: Reactive oxygen species
SKC: Na + transporter protein
SOD: Superoxide dismutases
TFs: Transcription factors
MAPK: Mitogen-activated protein kinase
